# Neutrophil Extracellular Traps Activate Meningeal Fibroblast to Aggravate Subarachnoid Fibrosis in Kaolin‐Induced Hydrocephalus in Rats

**DOI:** 10.1002/iid3.70268

**Published:** 2025-11-14

**Authors:** Chao Ma, Zhou Feng, Binyuan Xiong, Liang Liang, Shengyan Liu, Lingxia Min, Qiang Zhang, Peiwen Guo, Jingyu Chen, Liang Tan, Jingming Hou, Zhi Chen

**Affiliations:** ^1^ Department of Rehabilitation, Southwest Hospital Third Military Medical University (Army Medical University) Chongqing China; ^2^ Department of Emergency Affiliated Hospital of Zunyi Medical University Zunyi China; ^3^ Department of Neurosurgery, Southwest Hospital Third Military Medical University (Army Medical University) Chongqing China; ^4^ Chinese People's Liberation Army Western Theater General Hospital Chengdu China; ^5^ Chongqing Mental Health Center Chongqing China; ^6^ Department of Neurosurgery The 961th Hospital of the Chinese People's Liberation Army Joint Logistic Support Force Qiqihar China

**Keywords:** DNase I, fibroblast, fibrosis, hydrocephalus, neutrophil extracellular traps

## Abstract

**Background and Objectives:**

Subarachnoid fibrosis is the key pathology of hydrocephalus, but its underlying mechanisms remains poorly understood. In the present study, we aim to verify the hypothesis that neutrophil extracellular traps (NETs), released by neutrophils infiltrated into the subarachnoid space following hemorrhage and infection, might be a crucial culprits in promoting subarachnoid fibrosis in hydrocephalus.

**Methods:**

Firstly, NETs in cerebrospinal fluid (CSF) specimens from patients and subarachnoid fibrosis of kaolin‐induced hydrocephalus rat model were detected by assay kit and immunofluorescence, respectively. Secondly, kaolin‐induced hydrocephalus rats were treated by peptidylarginine deiminase 4 (PAD4) inhibitor and DNase I. NETs, subarachnoid fibrosis, reactive gliosis, proliferation and differentiation of meningeal fibroblasts were detected by immunofluorescence and Western Blot (WB), ventricular volumes were evaluated by magnetic resonance imaging (MRI). Finally, primary meningeal fibroblasts were stimulated with NET, and their proliferation and differentiation were measured by flow cytometer, WB and immunofluorescence, respectively.

**Results:**

Combining with CSF specimens from patients and hydrocephalus rat model, we found that infiltrating neutrophils release NETs in the subarachnoid space after subarachnoid hemorrhage and hydrocephalus. Further, combining with in vivo animal experiments and in vitro experiments, we demonstrated that NETs aggravate subarachnoid fibrosis to promote hydrocephalus via stimulating the proliferation and differentiation of meningeal fibroblasts. What′s more, both inhibiting NETs production with PAD4 inhibitor and degrading NETs with DNase I significantly prevent the development of hydrocephalus by attenuating subarachnoid fibrosis.

**Conclusion:**

NETs aggravate subarachnoid fibrosis to promote hydrocephalus via stimulating the proliferation and differentiation of meningeal fibroblasts, and restricting NETs significantly prevents the development of hydrocephalus through attenuating subarachnoid fibrosis. It indicates that NETs might be a promising potential target for clinical hydrocephalus treatment.

## Graphical abstract

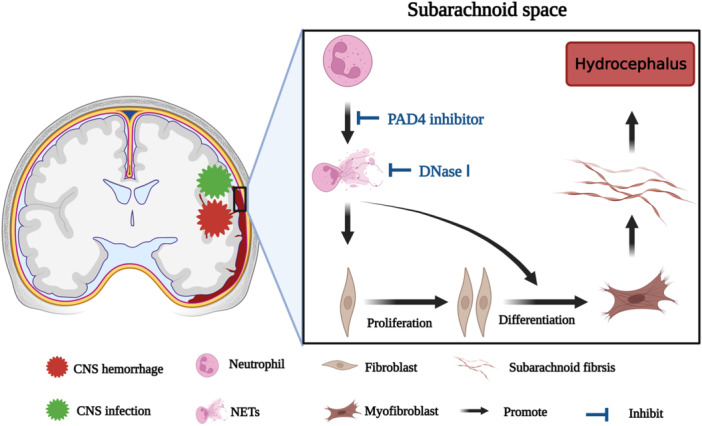

## 1 Introduction

Hydrocephalus is a prevalent neurosurgical condition marked by the enlargement of the cerebral ventricles [1]. As one of the most serious complications of multiple neurological diseases, it almost indicates a poor prognosis [2]. At present, the most widely accepted methods for treating hydrocephalus encompass the shunting of cerebrospinal fluid (CSF) and performing endoscopic third ventriculostomy [3, 4]. However, both methods are not ideal treatments as frequently occurred complications [3, 4]. Thus, it is urgent to further investigate the underlying pathogenesis of hydrocephalus, so as to propose more effective treatments.

Subarachnoid fibrosis is acknowledged as a common underlying cause of various types of hydrocephalus, particularly post‐hemorrhagic hydrocephalus (PHH) and post‐infectious hydrocephalus (PIH) [5, 6]. And targeting excessive fibrosis in the subarachnoid space (SAS) has achieved a certain degree of therapeutic effects [7, 8, 9]. However, the exact mechanism of subarachnoid fibrosis in hydrocephalus remains poorly understood, which might be the fundamental obstacle to developing more powerful treatments.

Neutrophils, as the crucial component of the innate immune system, infiltrating into the SAS after central nervous system (CNS) hemorrhage and infection [10, 11, 12, 13], which leads to subsequent subarachnoid inflammation [7, 14, 15]. In addition to releasing inflammatory mediators and recruiting inflammatory cells, such as monocytes/macrophages, neutrophils were found to produce neutrophil extracellular traps (NETs) after activation [16]. NETs were initially supposed to disarm and kill bacteria [16] but were subsequently proven to involve in multiple CNS disorders [17], including intracerebral hemorrhage [18] and spinal cord injury [19] demonstrated in our previous study. Moreover, recent studies indicated that NETs accelerate cardiac and pulmonary fibrosis by activating fibroblasts [20, 21]. These valuable findings proposed new perspectives into the underlying mechanisms driving fibrosis and were supposed to provide more efficient therapeutic approaches [22]. Attractively, recent studies also detected abundant NETs in the CSF after CNS infections [23, 24], which indicated that it may contribute to the subsequent fibrosis in hydrocephalus. However, whether NETs present in the SAS after intracranial hemorrhagic diseases (such as subarachnoid hemorrhage, SAH) likewise, and the precise function of NETs in the development of subarachnoid fibrosis linked to hydrocephalus has not been explored previously. Given the similarities between rats and humans in physiology and behavior, the use of rat models to explore the significance of NETs in subarachnoid fibrosis is of great importance

Therefore, we first detected NETs in the CSF of SAH patients and subsequently utilized an experimental model of kaolin‐induced hydrocephalus to explored whether NETs were implicated in subarachnoid fibrosis in hydrocephalus. Moreover, we further investigated the effect of restricting NETs on hydrocephalus.

## 2 Materials and Methods

### 2.1 Obtaining Patient Specimens

CSF was collected within 3 days of aneurysmal SAH onset via routine lumbar puncture. All patients received non‐contrast CT (NCCT) within 24 h of symptom onset. Inclusion criteria: (1) Spontaneous SAH confirmed on admission by CT or lumbar puncture; (2) Intracranial aneurysm verified as bleeding source via DSA/surgery; (3) Complete baseline thick‐slice NCCT and clinical data. Exclusion criteria: (1) Intracranial hemorrhage from non‐aneurysmal causes (trauma, hypertension, vascular malformation, or tumor); (2) Prior intracranial surgery. Control patients without hemorrhage but received lumbar puncture for diagnosis. Additional clinical characteristics of SAH patients are provided in Supporting Information S2: Table S1. After centrifuging at 500*g* for 5 min, the supernatant of CSF specimens was collected for quantification. Cell‐free DNA (cfDNA) in CSF specimens was determined by Quant‐iT Pico‐Green dsDNA kit (Invitrogen).

### 2.2 Rat Model and Treatment

Adult Sprague–Dawley rats (male, 250–300 g) were obtained from the Army Medical University Animal Center and housed under standard conditions (25 ± 2°C, the humidity of 55 ± 5%, and 12 h of circulating light). Animal experiments were performed in two parts. Hydrocephalus was induced through the injection of a kaolin suspension into the basal cistern. For the first part, the experimental rats were randomly assigned into three groups (sham, vehicle, and anti‐polymorphonuclear neutrophil (anti‐PMN), *n* = 10, respectively). Anti‐PMN group was injected with 0.3 mL rabbit anti‐rat‐PMN serum (Accurate Chemical & Scientific Corporation, intravenously injection) 24 h before hydrocephalus induction and last for 3 days to deplete neutrophil. The vehicle group was administered an equivalent volume of saline at the same time after hydrocephalus induction. For the second part, the experimental rats were randomly assigned into five groups (sham, dimethylsulfoxide [DMSO], Cl‐amidine, saline and DNaseI; *n* = 12, respectively). The Cl‐amidine group was injected with Cl‐amidine (MedChemExpress; 50 mg/kg; intraperitoneally) treatment daily after hydrocephalus induction for 3 days, while the DMSO group was administered an equivalent dose of 5% DMSO. DNaseI group received DNaseI (diluted in saline; Roche; 3500U; intraventricularly) [24] treatment daily after hydrocephalus induction for 3 days and the saline group received an equivalent volume of saline during the same period.

### 2.3 Communicating Hydrocephalus Model

As mentioned early [25], communicating hydrocephalus model was established by kaolin (Sigma‐Aldrich). The experimental rats were anesthetized with pentobarbital (40 mg/kg, intraperitoneal injection) and underwent exposure of the ventral atlanto‐occipital membrane through incision along the ventral midline of the neck. Subsequently, 30 µL sterilized suspension of 25% kaolin was slowly injected (a 29‐gauge needle) into the SAS of basal cistern (more than 10 s) for communicating hydrocephalus model establish and the sham group received an equivalent volume of saline instead of kaolin suspension. After the communicating hydrocephalus model established, the different group received corresponding drug administration for the following experiments. Three rats with abnormal behavioral activity and infections after surgery within 48 h were eliminated.

### 2.4 Intraventricular Injection

According to previous descriptions [26], intraventricular injection was performed. The experimental rats were anesthetized and fixed to the stereotactic frame. Then sterilized the scalp area and exposed the skull. A needle with a gauge of 29 was precisely positioned into the right lateral ventricle (LV) using stereotaxic techniques through the hole drilled on the skull (coordinates: posterior‐0.6 mm, lateral‐1.6 mm, and ventral to the bregma‐4.5 mm). For the Cl‐amidine group, Cl‐amidine (MedChemExpress; 50 mg/kg) solution was injected into the LV region at the rate of 2 µL/min. For the DNaseI group, DNaseI (diluted in saline; Roche; 3500U) solution was injected into the LV region. The DMSO and saline group injected the same volume of vehicle via the same route.

### 2.5 Magnetic Resonance Imaging Performance and Analysis

Rats were anesthetized with 2% isoflurane–air mixture during MRI examination at Day 28 after hydrocephalus induction. Images were acquired with a 7.0T MRI scanner (Bruker BioSpin) for measuring ventricular volumes. Coronal T2‐weighted images were acquired with the field view of 3.5 × 3.5 cm and 256 × 256 matrix, and total in 17 coronal slices were obtained (*n* = 6). The ventricular volumes were calculated as previously methods [8]. The Evans ratio was calculated by the ratio of the greatest width of both LVs to the greatest width of the brain with the coronal MRI scan at the level of Foramen of Monro.

### 2.6 Neutrophil Isolation and NETs Induction

Neutrophil isolation was conducted as previously described [27]. Collected the anticoagulated whole blood and mixed with 1% dextran. Then transfer the leukocyte‐rich top layer to another new tube and centrifuge at 500*g* for 10 min. Suspend the pellet in Hanks′ Balanced Salt Solution (HBSS) and loaded onto the top of Histopaque1077 and Histopaque1119 density gradients. After 30 min of centrifugation at 700*g*, neutrophils were harvested from the boundary between the Histopaque1077 and Histopaque1119 layers.

Isolated neutrophils were activated using phorbol myristate acetate (PMA, 500 nM; MedChemExpress) at 37°C for 4 h to induce NETs [28]. Then centrifugation at 200*g* for 5 min and the supernatant containing NETs was obtained. The concentration of NETs was detected using PicoGreen dsDNA assay kit as mentioned before.

### 2.7 Fibroblasts Cultures and Treatment

Primary fibroblasts were isolated from cortical meninges of postnatal Day 3 SD rats. After being carefully removed from the cortex, rat meninges were minced and dissociated in trypsin with collagenase (Invitrogen) at 37°C for 30 min with trituration. An equal volume of Dulbecco′s modified essential medium (DMEM)/F12 (Invitrogen) contained with FBS (10%) was added to terminate the digestion and pass through a 70‐μm cell strainer. The resulting flow‐through were collected and centrifuged at 500*g* for 5 min, then resuspended the cell pellet with 3 mL medium and repeated the centrifugation and resuspension twice. The isolated cells were resuspended in complete DMEM/F12 growth medium containing 10% fetal bovine serum (FBS) and 1% penicillin‐streptomycin. Following incubation under standard culture conditions, non‐adherent cells were removed through medium replacement at 72 h. Adherent cells were maintained as primary meningeal fibroblast cultures in growth medium until confluent, with medium refreshed every 48–72 h. The cultured cells were treated with unstimulated neutrophils (control group), inducted NETs (500 ng/mL, NETs group), or NETs pre‐treated with DNaseI (40 IE/mL) (NETs + DNaseI group) for 24 h. And then cells were collected for further experiments.

### 2.8 Immunofluorescence Staining

Experimental rats underwent sequential transcardiac perfusion with 0.01 M phosphate‐buffered saline (PBS) and 4% paraformaldehyde. Then brains were removed and fixed with 4% paraformaldehyde for 24 h. After dehydrated with 30% sucrose solution and embedded with optimal cutting temperature embedding matrix (Sakura Finetek USA Inc, Torrance, Calif), brain tissues were cut into 18‐µm‐thick coronal sections by cryostat microtome and the slices were stored at −20°C before staining. Similarly, cultured cells were fixed with 4% PFA. For the immunofluorescence staining, brain sections or cells were permeabilized using 0.3% TritonX‐100 in PBS. After being blocked with 10% goat serum in 5% BSA, the samples were incubated with primary antibodies overnight at 4°C. After washed with PBS, the corresponding secondary antibodies were incubated for 2 h at 37°C and label the cell nucleus with DAPI. The stained sections were visualized and captured by a confocal fluorescent microscope (LSM880, Zeiss) using a ×10 or ×20 objective under identical setting. Images were taken of the ventral SAS at the level of the bregma for subarachnoid fibrosis evaluation, and the corpus callosum and periventricular area for astrocytosis evaluation. The Lamin, Fibronectin and GFAP IF staining results were evaluated using ImageJ analysis software (National Institutes of Health) as described previously [7]. The primary antibodies used in this study as follows: anti‐Histone H3 (citrulline R2 + R8 + R17) (rabbit, 1:500, Abcam, ab281584), anti‐Myeloperoxidase (MPO, mouse, 1:50, Abcam, ab90810), anti‐glial fibrillary acidic protein (GFAP, mouse, 1:500, Biosensis, M‐1375‐100), anti‐Laminin (rabbit, 1:500, Dako, Z0097), anti‐CD68 (rabbit, 1:200, Abcam, ab283654), anti‐fibronectin (mouse, 1:500, Abcam, ab6328), anti‐fibroblast‐specific protein‐1 (FSP1, rabbit, 1:200, Invitrogen, MA5‐32347), anti‐α‐smooth muscle actin (α‐SMA, mouse, 1:100, Invitrogen, MA1‐06110), anti‐proliferating cell nuclear antigen (PCNA, mouse, 1:500, GeneTex, GTX20029).

SYTOX Orange was stained by the previous method [29]. Briefly, samples were incubated with SYTOX Orange (5 mM, Molecular Probes) for 10 min. After washing with PBS, the samples were visualized and captured by confocal fluorescent microscope (LSM880, Zeiss).

### 2.9 Western Blot (WB)

WB was performed as described [30]. Briefly, samples were obtained from the ventral SAS at the level of the bregma or cultured cells were collected for WB analysis. And the samples were lysed with RIPA buffer contained with protease and phosphatase inhibitors for 15 min on ice. Protein extract was obtained through centrifuge at 13,000*g* for 15 min. Subsequently, each group of protein extract concentration were measured by Bicinchoninic Acid Protein Assay kit (Thermo Scientific, Pittsburgh, Penn) and equal quantities of each sample (40 µg) were loaded and electrophoresis through SDS‐PAGE. The separated protein gel subsequently transferred onto PVDF membranes and blocked using 5% BSA in PBST for 2 h at room temperature. After blocking the membranes were incubated with primary antibodies at 4°C overnight. After washing with PBST the appropriate secondary antibodies were incubated for 2 h at room temperature. Finally, the protein bands were detected and imaged utilizing a Fusion Edge instrument (Vilber). The primary antibodies are as follows: anti‐Histone H3 (citrulline R2 + R8 + R17) (rabbit, 1:500, Abcam, ab281584), anti‐FSP1 (rabbit, 1:200, Invitrogen, MA5‐32347), anti‐PCNA (mouse, 1:500, GeneTex, GTX20029), anti‐α‐SMA (mouse, 1:400, Invitrogen, MA1‐06110), anti‐β‐tubulin (rabbit, 1:1000, Zen‐bio, R23623), anti‐GAPDH (rabbit, 1:1000, Zen‐bio, R380646).

### 2.10 Fibroblast Proliferation Assays

To evaluate the proliferation of cultured fibroblasts after different treatments, 5‐ethynyl‐2′‐deoxyuridine (EdU, 20 μM) was added to cultured fibroblasts for 4 h before termination of the cultures. Cells labeled with EdU^+^ cells were subsequently identified through flow cytometer (FACSuite, BD Biosciences) utilizing the Click‐iT EdU Cell Proliferation Kit with Alexa Fluor 488 (Beyotime).

### 2.11 Statistical Analysis

Data were processed by Prism v8.0 software (GraphPad) and were presented as means ± standard deviation. To compare the two groups, Student′s *t*‐test was employed. To compare the multi‐groups, one‐way analysis of variance (ANOVA) followed by Bonferroni post hoc test was employed. *p* < 0.05 was considered statistically significant.

## 3 Results

### 3.1 NETs Are Present in the CSF of SAH Patients

**Figure 1:**
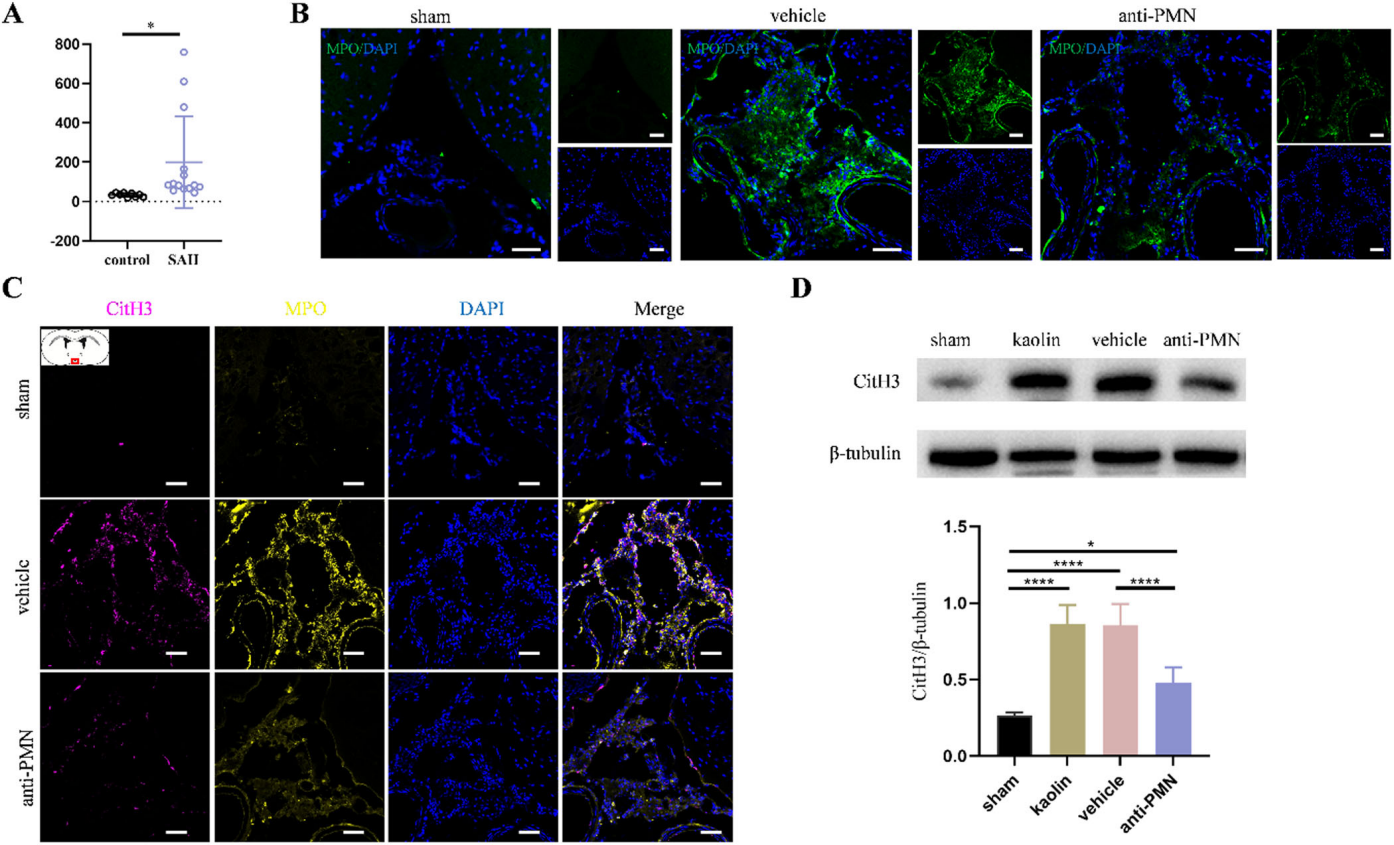
Neutrophils infiltrate into the subarachnoid space to produce NETs after SAH and kaolin‐induced hydrocephalus. (A) Cell‐free DNA (cfDNA) concentration in the CSF of control patients (*n* = 9) and SAH patients (*n* = 14). (B) Representative images of MPO (green) positive neutrophils in the subarachnoid space from corresponding groups 3 days after hydrocephalus induction. Scale bars = 50 μm. (C) Representative images of CitH3 (magenta) and MPO (yellow) marked NETs in the subarachnoid space from corresponding groups 3 days after hydrocephalus induction, the schematic illustration (top left) shows sections taken at the level of the Bregma. Scale bars = 50 μm. (D) Representative western blot images and quantification of CitH3 in the subarachnoid space from corresponding groups 3 days after hydrocephalus induction (*n* = 6). Data shown as means ± SD; *p < 0.05, ****p < 0.0001.

As PHH and PIH are two main forms of hydrocephalus and share some pivotal pathophysiological mechanisms [4]. And the presence of NETs in the CSF of patients with CNS infections were documented recently [23, 24]. Thus, it prompted us to investigate whether NETs were also present in the CSF of patients with SAH. We detected NETs via quantification of cfDNA and the result demonstrated that cfDNA was increased in the CSF samples from SAH patients compared to the control group (Figure 1A). These findings suggest that NETs may probably involve in the pathophysiological process of PHH and PIH.

### 3.2 Neutrophils Infiltrate Into SAS and Produce NETs in Kaolin‐Induced Hydrocephalus

To simulate the common pathological subarachnoid fibrosis of PHH and PIH, we established the kaolin‐induced hydrocephalus. The presence of neutrophils in the SAS after kaolin‐induced hydrocephalus was reported previously [7]. Similarly, Immunofluorescence staining results showed that neutrophils infiltrated from the arteries into the SAS (Figure 1B, Supporting Information S1: Figure 1A), which subsequently produced NETs 3 days after hydrocephalus induction (Figure 1D,E). Then we used anti‐rat PMN serum to effectively deplete neutrophils (Supporting Information S1: Figure 1B) and found that both neutrophils and NETs were reduced in the SAS (Figure 1B–D). These results demonstrated that circulating neutrophils are recruited into SAS to produce NETs in hydrocephalus.

**Figure 2:**
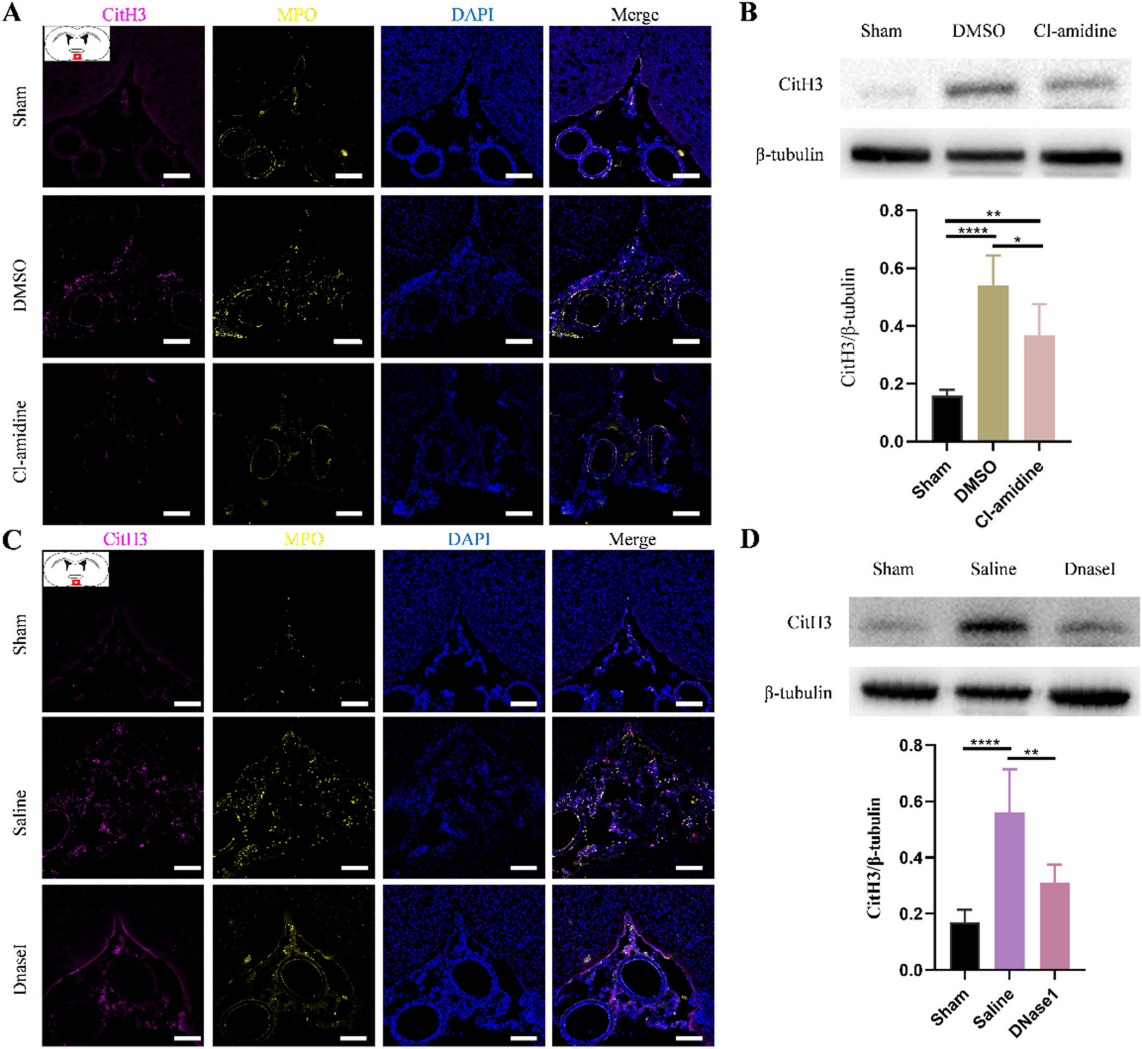
NETs are restricted by PAD4 inhibitor and DNaseI after kaolin‐induced hydrocephalus. (A) Representative images of CitH3 (magenta) and MPO (yellow) marked NETs in the subarachnoid space from sham, DMSO, and Cl‐amidine groups 3 days after hydrocephalus induction. Scale bars = 100 μm. (B) Representative western blot images and quantification of the CitH3 levels in the subarachnoid space from sham, DMSO, and Cl‐amidine groups 3 days after hydrocephalus induction. Data shown as means ± SD; *n* = 6; *p < 0.05, ****p < 0.0001. (C) Representative images of CitH3 (magenta) and MPO (yellow) marked NETs in the subarachnoid space from sham, saline, and DNaseI groups 3 days after hydrocephalus induction. Scale bars = 100 μm. (D) Representative western blot images and quantification of the CitH3 levels in the subarachnoid space from sham, saline, and DNaseI groups 3 days after hydrocephalus induction. Data shown as means ± SD; *n* = 6; *p < 0.05, **p < 0.01, ****p < 0.0001.

To determine whether inhibiting peptidylarginine deiminase 4 (PAD4) and degrading extracellular DNA network effectively restrict NETs in hydrocephalus, we performed Cl‐amidine (a PAD4 inhibitor) and DNaseI treatment. Consistent with previously reported in other CNS disorders [19, 31], we observed that both Cl‐amidine and DNaseI treatment significantly restricted the level of NETs in the SAS after hydrocephalus (Figure 2A,C) and the decreased protein level of CitH3 upon Cl‐amidine and DNaseI treatment further confirm it (Figure 2B,D).

### 3.3 Restricting NETs Attenuates Fibrosis in the SAS

**Figure 3:**
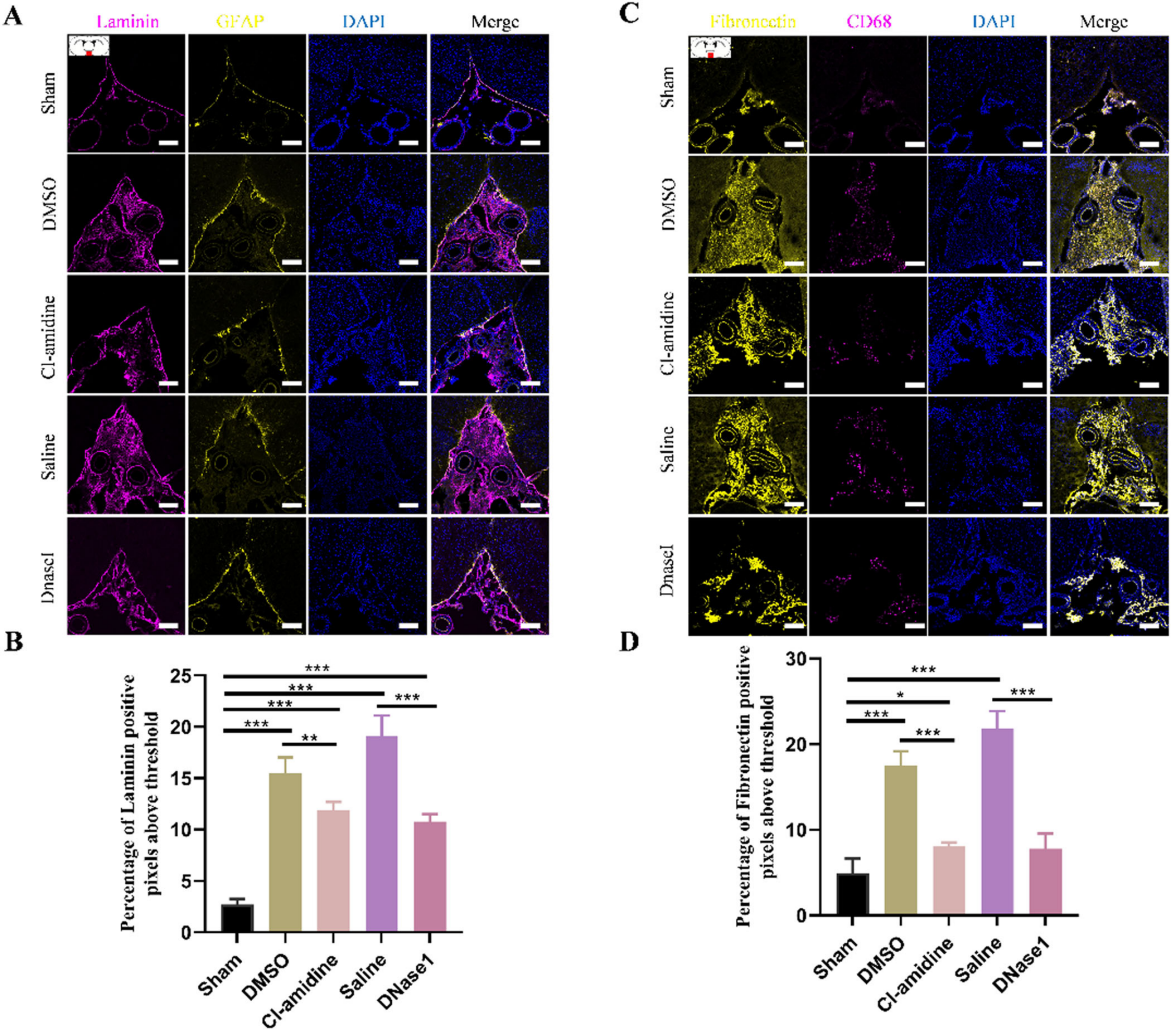
Restricting NETs attenuates fibrosis in the subarachnoid space after kaolin‐induced hydrocephalus. (A) Representative images of laminin (magenta) deposition along with GFAP‐positive (yellow) astrocytes penetrating fibrotic areas in the subarachnoid space from DMSO and saline groups 28 days after hydrocephalus induction, which was attenuated by both Cl‐amidine and DNaseI. (B) Quantification of Laminin‐positive staining in the subarachnoid space. (C) Representative images of fibronectin (yellow) deposition along with CD68‐positive (magenta) macrophages infiltrating in the subarachnoid space from DMSO and saline groups 28 days after hydrocephalus induction, which was attenuated by both Cl‐amidine and DNaseI. (D) Quantification of fibronectin‐positive staining in the subarachnoid space. Scale bars = 100 μm. Data shown as means ± SD; *n* = 6; *p < 0.05, **p < 0.01, ***p < 0.001.

To test whether NETs contribute to subarachnoid fibrosis in hydrocephalus, we evaluated the deposition of extracellular matrixes in the SAS 28 days after hydrocephalus induction. Immunofluorescence staining results indicated that laminin deposited throughout the SAS after hydrocephalus (Figure 3A,B). Likewise, the deposition of fibronectin also extended throughout the SAS after hydrocephalus, which dotted with abundant inflammatory macrophages (Figure 3C,D). While restricting NETs with both Cl‐amidine and DNaseI attenuated extracellular matrixes deposition in the SAS (Figure 3A–D). These findings demonstrated that restricting NETs significantly attenuates subarachnoid fibrosis in hydrocephalus.

### 3.4 Restricting NETs Prevents the Development of Hydrocephalus

**Figure 4:**
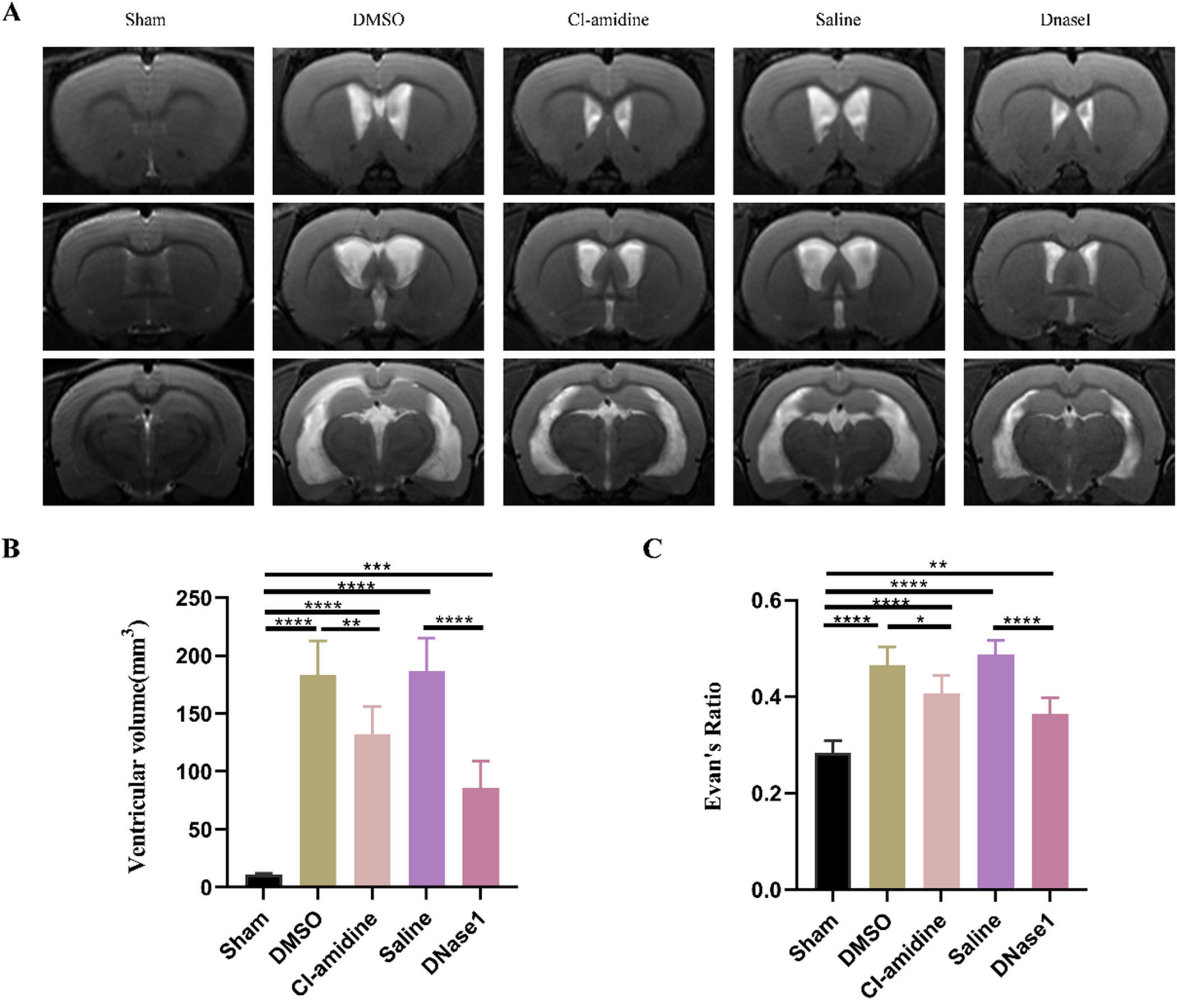
Restricting NETs prevents ventricular enlargement of kaolin‐induced hydrocephalus. (A) Representative T2‐weighted MRI images of sham, DMSO, Cl‐amidine, saline, and DNaseI groups 28 days after hydrocephalus induction. (B) Ventricular volumes and (C) Evans ratio in the five groups 28 days after hydrocephalus induction. Data shown as means ± SD; *n* = 6; *p < 0.05, **p < 0.01, ***p < 0.001, ****p < 0.0001.

We next evaluated hydrocephalus by measuring ventricular volume and Evans ratio on Day 28 after hydrocephalus induction. MRI results demonstrated a significantly enlarged of ventricular volume following kaolin injection. However, this was effectively attenuated by Cl‐amidine and DNaseI treatment (Figure 4A,B). Similarly, the Evans ratio was increased after hydrocephalus induction, which was reduced by both Cl‐amidine and DNaseI (Figure 4A,C). These findings indicate that restricting NETs effectively attenuated the development of hydrocephalus.

### 3.5 Restricting NETs Reduced Reactive Gliosis

**Figure 5:**
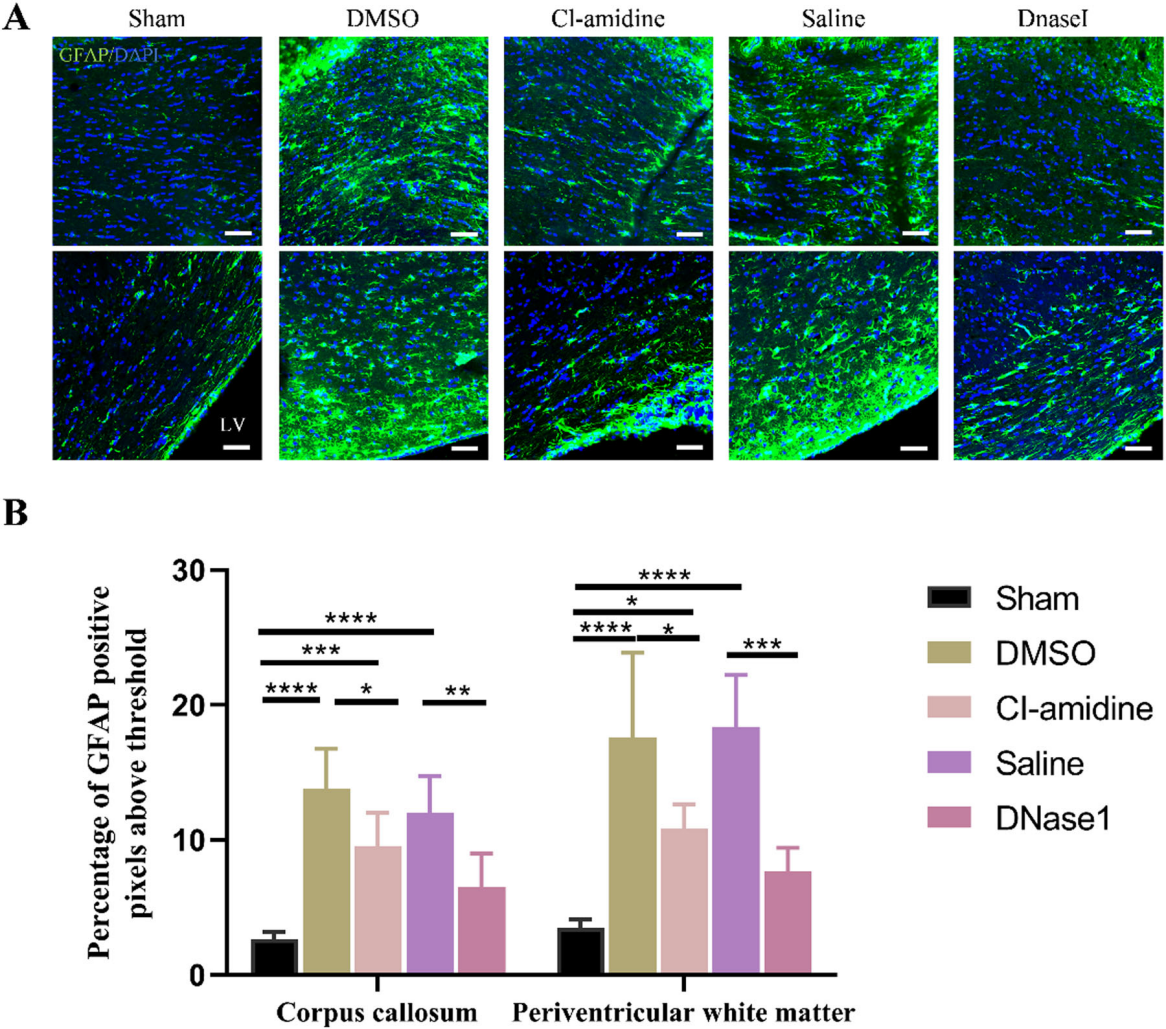
Restricting NETs reduces hydrocephalus‐induced reactive astrocytosis. (A) Representative images of GFAP‐positive (green) astrocytes in the corpus callosum (upper) and the periventricular area (lower) in the sham, DMSO, Cl‐amidine, saline, and DNaseI groups 28 days after hydrocephalus induction. Scale bars = 50 μm. (B) Histogram of the percentage of GFAP positive staining in the corpus callosum and periventricular area in the five groups. Data shown as means ± SD; *n* = 6; *p < 0.05, **p < 0.01, ***p < 0.001, ****p < 0.0001.

To evaluate hydrocephalus‐induced brain tissue damage, we performed a reactive gliosis examination. Immunofluorescence staining results demonstrated that GFAP‐positive astrocytes were activated in both the corpus callosum and the periventricular area after hydrocephalus induction (Figure 5A,B). And both Cl‐amidine and DNaseI treatments reduced reactive astrocytosis (Figure 5A,B). These findings demonstrated that restricting NETs attenuates hydrocephalus‐induced brain tissue damage.

### 3.6 NETs Promote Proliferation and Differentiation of Meningeal Fibroblasts to Aggravate Subarachnoid Fibrosis

**Figure 6:**
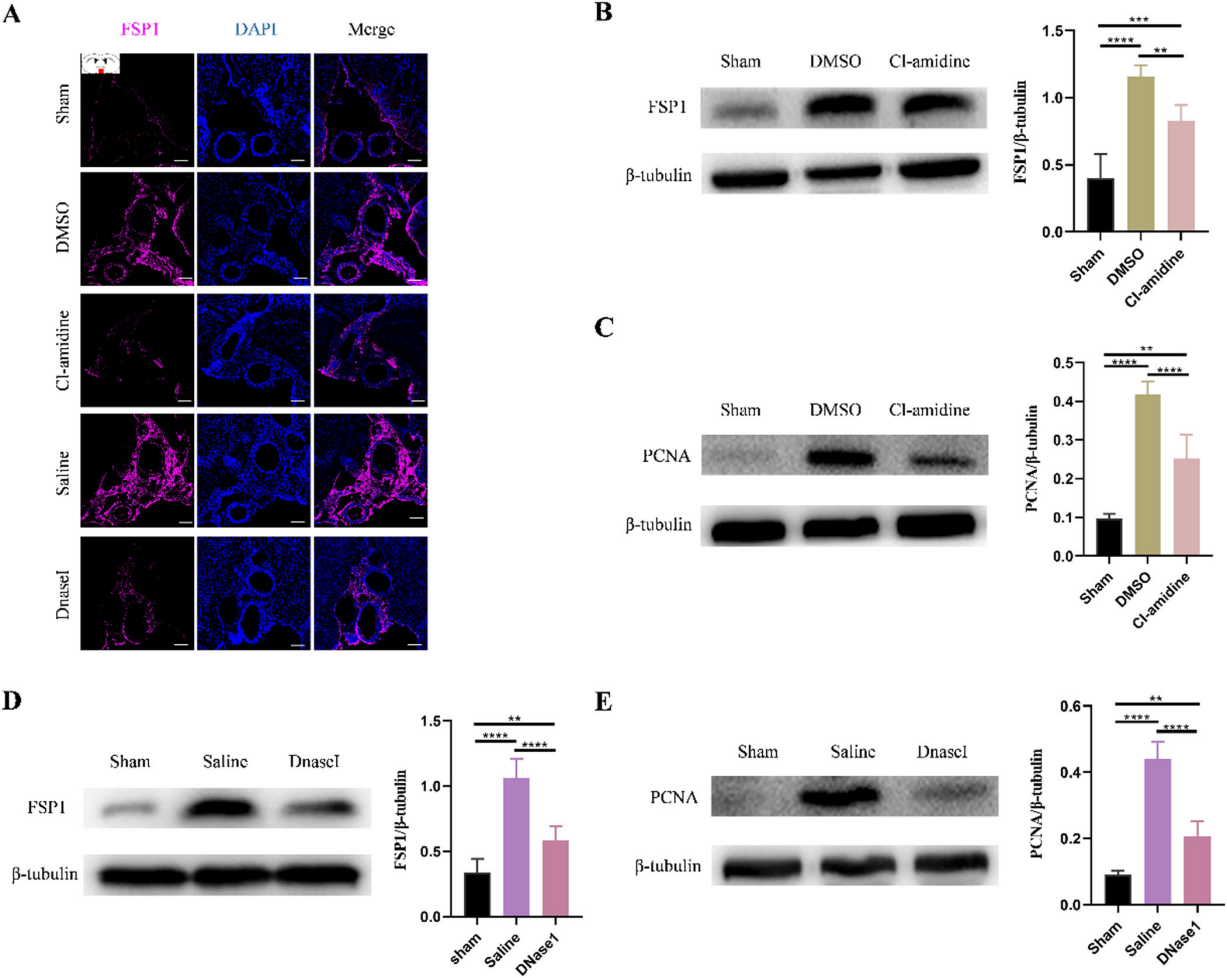
NETs promote the proliferation of meningeal fibroblasts after kaolin‐induced hydrocephalus. (A) Representative images of FSP1 (magenta) marked meningeal fibroblasts in the subarachnoid space from sham, DMSO, and Cl‐amidine, saline, and DNaseI groups 3 days after hydrocephalus induction. Scale bars = 100 μm. Representative western blot images and quantification of the FSP1 levels (B) and PCNA levels (C) in the subarachnoid space from sham, DMSO, and Cl‐amidine groups 3 days after hydrocephalus induction. Representative western blot images and quantification of the FSP1 levels (D) and PCNA levels (E) in the subarachnoid space from sham, saline, and DNaseI groups 3 days after hydrocephalus induction. Data shown as means ± SD; *n* = 6; **p < 0.01, ***p < 0.001, ****p < 0.0001.

**Figure 7:**
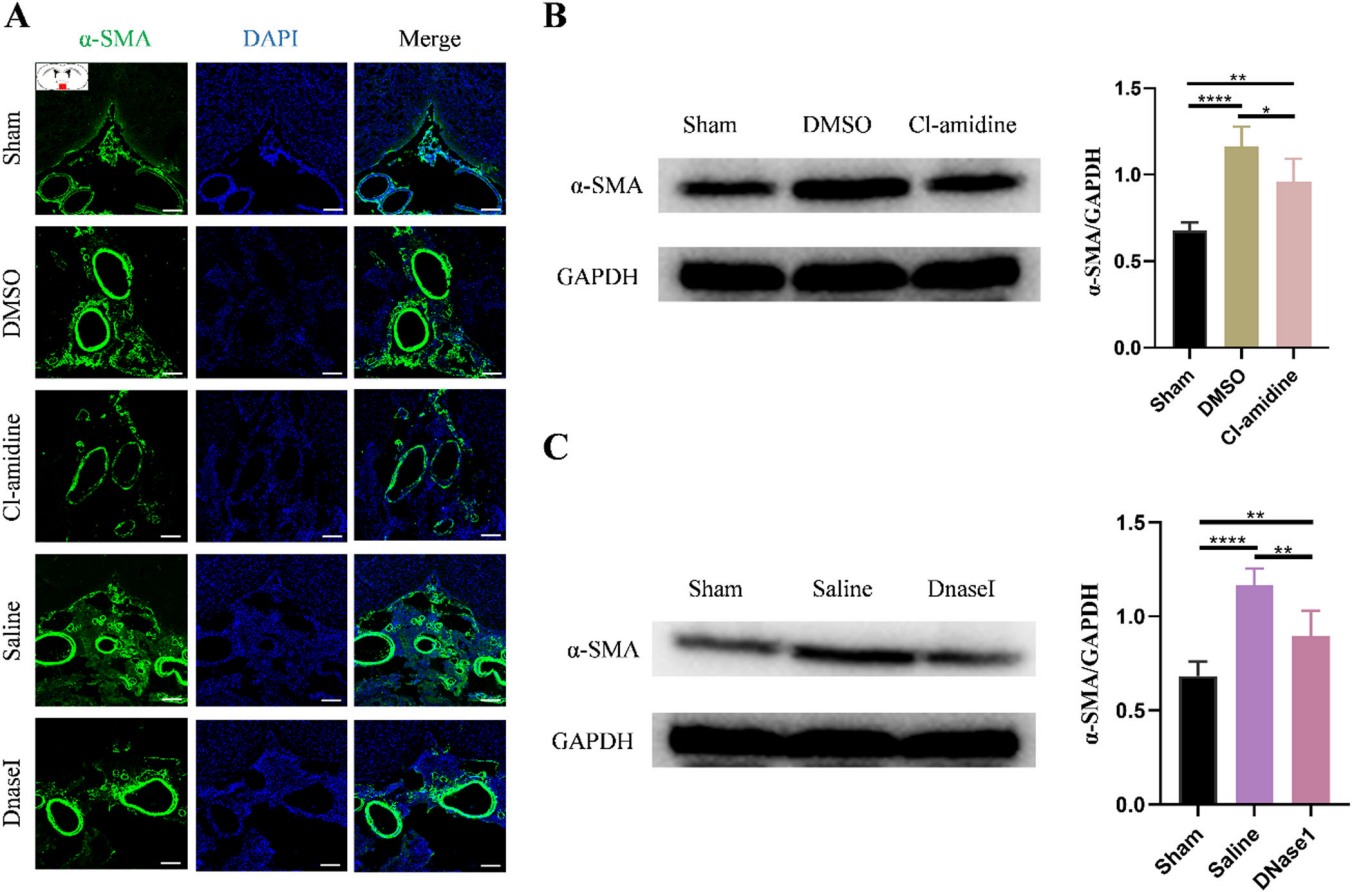
NETs promote differentiation of meningeal fibroblasts after kaolin‐induced hydrocephalus. (A) Representative images of α‐SMA (green) marked myofibroblast in the subarachnoid space from sham, DMSO, and Cl‐amidine, saline, and DNaseI groups 3 days after hydrocephalus induction. Scale bars = 100 μm. Representative western blot iamges and quantification of the α‐SMA levels in the subarachnoid space from sham, DMSO, and Cl‐amidine groups (B) and from sham, saline, and DNaseI groups (C) 3 days after hydrocephalus induction. Data shown as means ± SD; *n* = 6; *p < 0.05, **p < 0.01, ****p < 0.0001.

**Figure 8:**
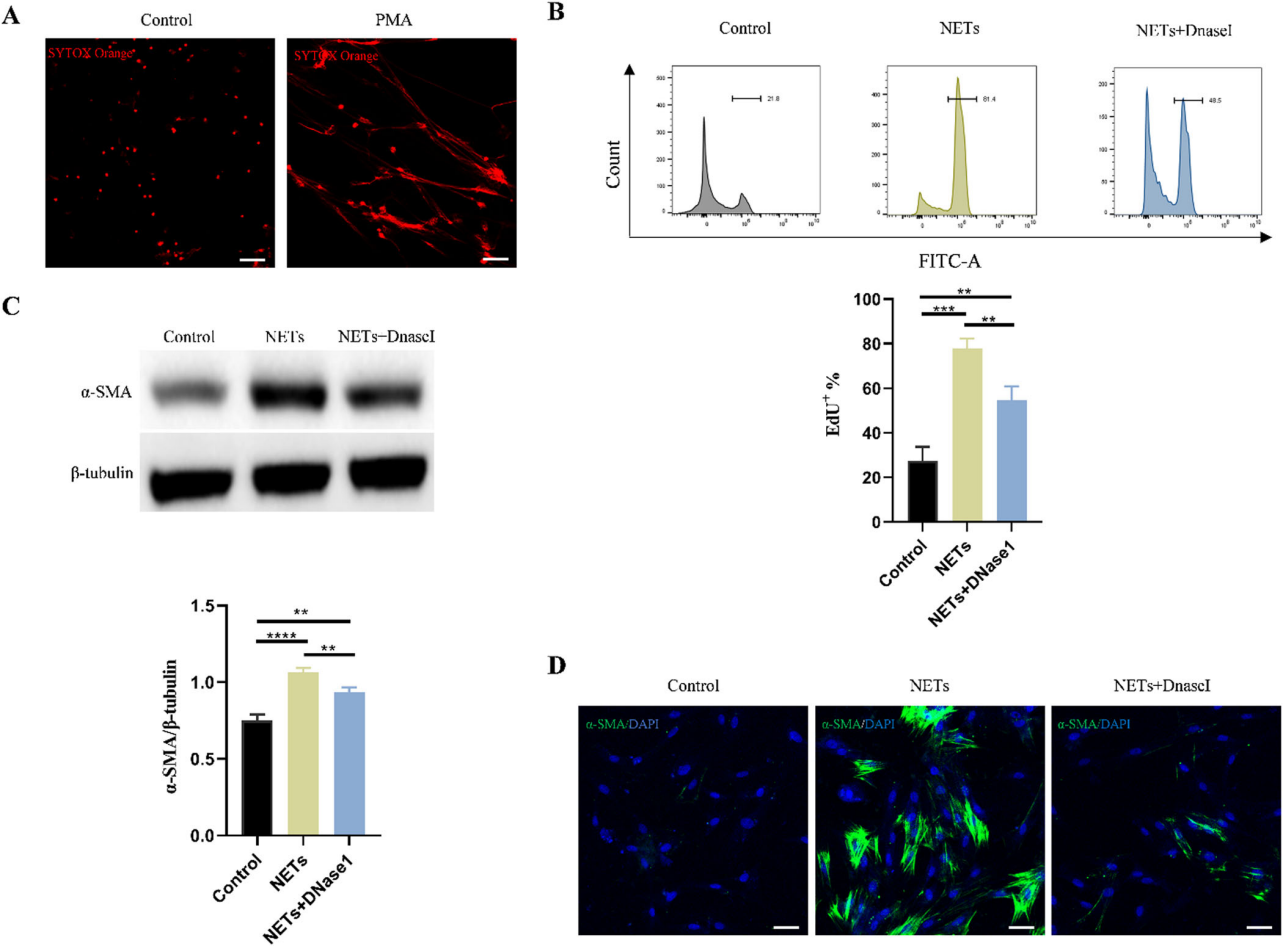
NETs promote proliferation and differentiation of meningeal fibroblasts in vitro. (A) Representative images of NETs induced by PMA that stained SYTOX Orange (red). Scale bars = 100 μm. (B) The proliferation of meningeal fibroblasts in control, NETs, and NETs + DNase I groups was detected by the EdU experiment using flow cytometry analysis. (C) Representative western blot images and quantification of the α‐SMA levels meningeal fibroblasts from control, NETs, and NETs + DNaseI groups. (D) Representative images of α‐SMA (Green) marked myofibroblast in control, NETs, and NETs + DNaseI groups. Scale bars = 50 μm. Data shown as means ± SD; *n* = 3; **p < 0.01, ***p < 0.001, ****p < 0.0001.

Finally, we explored how NETs aggravate subarachnoid fibrosis in hydrocephalus. Three days after hydrocephalus induction, we performed immunofluorescence staining and the results revealed that NETs significantly upregulated the expression of fibroblast‐specific protein 1 (Fsp1), a meningeal fibroblast marker (Figure 6A). Conversely, NETs inhibition via Cl‐amidine and DNaseI treatment markedly reduced the Fsp1 expression (Figure 6A), suggesting that NETs may promote meningeal fibroblast proliferation. To further validate these findings, we detected the protein level of FSP1 and PCNA in SAS tissue. Consistent with the immunofluorescence data, NETs were found to promote meningeal fibroblast proliferation, whereas Cl‐amidine and DNaseI treatment effectively attenuated this proliferative response following hydrocephalus induction (Figure 6B–E). These findings indicate that treatment with Cl‐amidine and DNaseI could attenuated the meningeal fibroblast proliferation after hydrocephalus induction (Figure 6A–E). Furthermore, we detected the differentiation of meningeal fibroblasts and found that NETs promote meningeal fibroblasts differentiation into myofibroblast (Figure 7A) in the SAS. While restricting NETs by Cl‐amidine and DNaseI significantly attenuated the differentiation of meningeal fibroblasts (Figure 7B,C). These results suggest that NETs promote meningeal fibroblasts proliferation and differentiation into myofibroblast after hydrocephalus induction and restriction of NETs could alleviate it. To further confirm whether NETs promote proliferation and differentiation of meningeal fibroblasts in vitro, we treated primary meningeal fibroblasts with NETs (Figure 8A). Consistent with results we observed in vivo, NETs significantly promoted proliferation and differentiation of meningeal fibroblasts, which was attenuated by DNaseI (Figure 8B–D). These findings indicate that NETs promote the proliferation and differentiation of meningeal fibroblasts to aggravate subarachnoid fibrosis in hydrocephalus.

## 4 Discussion

**Figure 9:**
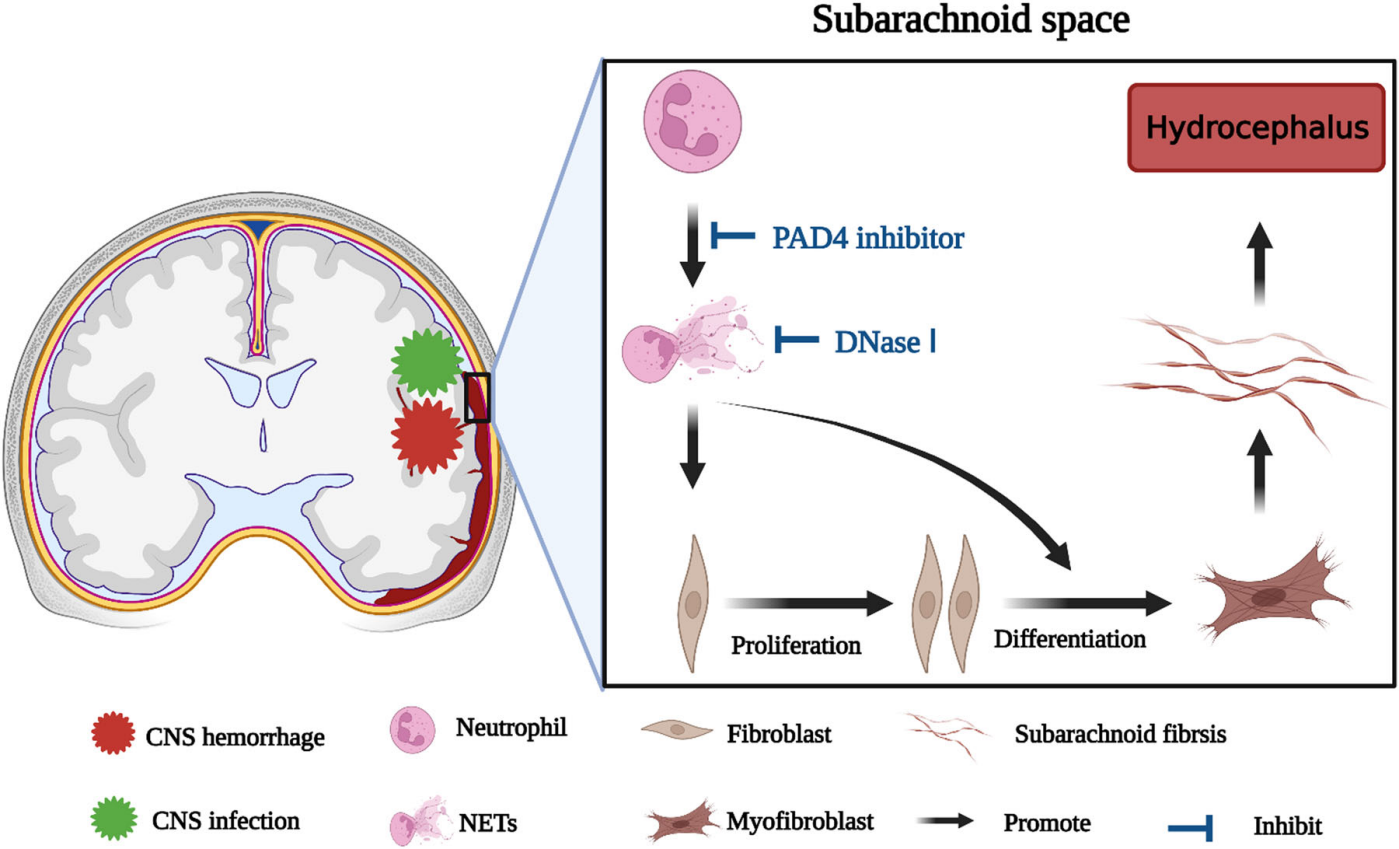
The schematic diagram for NETs‐mediated subarachnoid fibrosis to aggravate hydrocephalus after multiple CNS disorders. After CNS hemorrhage or infection, neutrophils infiltrate into the subarachnoid space to release NETs, which stimulate the proliferation and differentiation of meningeal fibroblasts to promote subarachnoid fibrosis, thus aggravating hydrocephalus. While inhibiting NETs formation with a PAD4 inhibitor or degrading NETs with DNaseI prevents the development of hydrocephalus through attenuating subarachnoid fibrosis.

Our study first found that NETs are present in both human CSF after SAH and SAS of kaolin‐induced communicating hydrocephalus rats. Moreover, we demonstrated that NETs aggravate subarachnoid fibrosis to promote hydrocephalus by stimulating the proliferation and differentiation of meningeal fibroblasts. Furthermore, restricting NETs with either Cl‐amidine or DNaseI significantly prevents the development of hydrocephalus through attenuating subarachnoid fibrosis (Figure 9).

Hemorrhage and infection in the CNS are the major causes of hydrocephalus [4], both of which are accompanied by neutrophil infiltration into the SAS [10, 11, 12]. But the role and mechanism of neutrophils in the occurrence and development of hydrocephalus remain unclear. In 2004, Brinkmann et al. [16] described a novel mechanism by which neutrophils are involved in pathophysiology, that activated neutrophils release decondensed chromatin, cytosolic, and granule proteins to form web‐like structures‐NETs. Recent studies reported that infiltrated neutrophils release NETs in the SAS to obstruct the CSF flow after CNS infection [23, 24]. Although in a previous study, we found that neutrophils infiltrate into the injured brain tissue to release NETs to aggravate injury after intracerebral hemorrhage [18], whether NETs would be involved in the pathology of hydrocephalus remains unclear. Therefore, we collected CSF samples from SAH patients to detect NETs via quantification of cfDNA and found that the level of NETs was relatively high compared to the control group. These results suggest that NETs may be closely related to hydrocephalus.

To further elucidate the relationship between NETs and the occurrence and development of hydrocephalus, we decided to establish a hydrocephalus model for verification. Kaolin‐induced hydrocephalus is a prevalent experimental model of hydrocephalus with the advantage of being stable and easy to induce [1]. What′s more, kaolin‐induced hydrocephalus was reported to effectively simulate some common pathogenesis of different forms of clinical hydrocephalus [7]. In agreement with the clinical results above, we found that NETs presented in SAS after kaolin‐induced hydrocephalus, which further indicates the inextricable relationship between NETs and hydrocephalus. Moreover, we found that subarachnoid NETs were significantly reduced by neutrophil depletion, indicating that infiltrating neutrophils were the main source of NETs. But we did not further observe the therapeutic effect of neutrophil depletion on hydrocephalus for that global neutrophil depletion may lead to an increased risk of infection, limiting clinical transformation [31].

Subarachnoid fibrosis is increasingly recognized as a significant pathological mechanism of various hydrocephalus, which contributes to the adhesion of SAS, impeding the circulation and absorption of CSF [7]. Corroborating the findings of another study [7], our previous studies demonstrated that subarachnoid fibrosis could be a promising therapeutic target for improving hydrocephalus. Meanwhile, our studies confirmed that kaolin‐induced hydrocephalus mimics subarachnoid fibrosis [8, 9]. Intriguingly, previous studies have indicated that NETs are crucial in the initiation and advancement of fibrosis in multiple systems [32]. In alignment with these studies, our present results show that inhibition of NETs formation by PAD4 inhibitor Cl‐amidine or the degradation of NETs with DNaseI both could alleviate subarachnoid fibrosis, indicating that NETs contribute to subarachnoid fibrosis after hydrocephalus. Furtherly, we found that restricting NETs with both PAD4 inhibitor and DNaseI ameliorate ventricular dilatation after kaolin‐induced hydrocephalus. These results suggest that NETs are a key culprit of hydrocephalus.

The proliferation and differentiation of fibroblasts is the main mechanism of organ and tissue fibrosis. Activated fibroblasts differentiate into myofibroblasts to produce amounts of extracellular matrix, which at a rate contributes to the development of fibrosis. Similarly, subarachnoid meningeal fibroblasts are activated after CNS hemorrhage or infectious disease, resulting in subarachnoid fibrosis. Our present study found that meningeal fibroblasts proliferated significantly and differentiated into myofibroblasts after kaolin‐induced hydrocephalus. As expected, restricting NETs significantly attenuated the proliferation and differentiation of meningeal fibroblasts, thus alleviating subarachnoid fibrosis. In line with these observations in vivo, our further in vitro experiments also demonstrated that NETs exert a prominent role in the proliferation and differentiation of fibroblast.

## 5 Limitations

There are some limitations in our present study. First, we did not follow up the subsequent development of hydrocephalus in these patients with SAH in the clinical research section. In the following studies, we will observe the correlation between NETs content in CSF and hydrocephalus in SAH patients, so as to further clarify the crucial role of NETs in the occurrence of hydrocephalus. Second, despite the presence of a variety of cell types in the SAS, we did not deeply explore the molecular mechanism of NETs promoting fibroblast proliferation and differentiation. We are planning to use multi‐omics methods to elucidate the underlying mechanisms of NETs regulation of fibroblasts to find new therapeutic targets for hydrocephalus.

## Author Contributions

**Chao Ma:** conceptualization, data curation, investigation, methodology, writing – original draft. **Zhou Feng:** data curation, investigation, methodology, visualization, writing – original draft. **Binyuan Xiong:** resources. **Liang Liang:** resources. **Shengyan Liu:** formal analysis, validation. **Lingxia Min:** formal analysis, methodology, validation. **Qiang Zhang:** validation. **Peiwen Guo:** validation. **Jingyu Chen:** formal analysis. **Liang Tan:** methodology, supervision. **Jingming Hou:** conceptualization, project administration, resources, supervision, writing – review and editing. **Zhi Chen:** conceptualization, funding acquisition, project administration, resources, writing – review and editing.

## Ethics Statement

The clinical study was granted approval by the Ethics Committee of the First Affiliated Hospital of the Army Medical University and adhered to the guidelines established in the Declaration of Helsinki. All experimental procedures were carried out in compliance with the animal use guidelines, which had been approved by the Army Medical University Animal Ethics Committee (AMUWEC20203820).

## Consent

Participants or their legal representatives have given written informed consent.

## Conflicts of Interest

The authors declare no conflicts of interest.

## Supporting information


**Supplementary Fig.1:** Neutrophils infiltrated from the arteries into the subarachnoid space to produce NETs.

**Table 1:** Demographic and clinical characteristics of SAH patients

## Data Availability

The data from this study can be obtained by the corresponding author upon a reasonable request.

## References

[1] D. Orešković and M. Klarica , “Development of Hydrocephalus and Classical Hypothesis of Cerebrospinal Fluid Hydrodynamics: Facts and Illusions,” Progress in Neurobiology 94, no. 3 (2011): 238–258, 10.1016/j.pneurobio.2011.05.005. 21641963

[2] Q. Chen , Z. Feng , Q. Tan , et al., “Post‐Hemorrhagic Hydrocephalus: Recent Advances and New Therapeutic Insights,” Journal of the Neurological Sciences 375 (2017): 220–230, 10.1016/j.jns.2017.01.072. 28320134

[3] K. T. Kahle , A. V. Kulkarni , D. D. Limbrick, J.r , and B. C. Warf , “Hydrocephalus in Children,” Lancet (London, England) 387, no. 10020 (2016): 788–799, 10.1016/s0140-6736(15)60694-8. 26256071

[4] J. K. Karimy , B. C. Reeves , E. Damisah , et al., “Inflammation in Acquired Hydrocephalus: Pathogenic Mechanisms and Therapeutic Targets,” Nature Reviews Neurology 16, no. 5 (2020): 285–296, 10.1038/s41582-020-0321-y. 32152460 PMC7375440

[5] O. Motohashi , M. Suzuki , N. Shida , et al., “Subarachnoid Haemorrhage Induced Proliferation of Leptomeningeal Cells and Deposition of Extracellular Matrices in the Arachnoid Granulations and Subarachnoid Space. Immunhistochemical Study,” Acta Neurochirurgica 136, no. 1–2 (1995): 88–91, 10.1007/bf01411441. 8748833

[6] S. Chatterjee and U. Chatterjee , “Overview of Post‐Infective Hydrocephalus,” Child′s Nervous System: ChNS: Official Journal of the International Society for Pediatric Neurosurgery 27, no. 10 (2011): 1693–1698, 10.1007/s00381-011-1557-z. 21928033

[7] H. Botfield , A. M. Gonzalez , O. Abdullah , et al., “Decorin Prevents the Development of Juvenile Communicating Hydrocephalus,” Brain: A Journal of Neurology 136, no. Pt 9 (2013): 2842–2858, 10.1093/brain/awt203. 23983032 PMC13375275

[8] Z. Feng , Q. Tan , J. Tang , et al., “Intraventricular Administration of Urokinase as a Novel Therapeutic Approach for Communicating Hydrocephalus,” Translational Research: the Journal of Laboratory and Clinical Medicine 180 (2017): 77–90, 10.1016/j.trsl.2016.08.004. 27614013

[9] Z. Feng , S. Liu , Q. Chen , et al., “uPA Alleviates Kaolin‐Induced Hydrocephalus by Promoting the Release and Activation of Hepatocyte Growth Factor in Rats,” Neuroscience Letters 731 (2020): 135011, 10.1016/j.neulet.2020.135011. 32497735

[10] J. J. Provencio , X. Fu , A. Siu , P. A. Rasmussen , S. L. Hazen , and R. M. Ransohoff , “CSF Neutrophils Are Implicated in the Development of Vasospasm in Subarachnoid Hemorrhage,” Neurocritical Care 12, no. 2 (2010): 244–251, 10.1007/s12028-009-9308-7. 19967568 PMC2844469

[11] T. Gris , P. Laplante , P. Thebault , et al., “Innate Immunity Activation in the Early Brain Injury Period Following Subarachnoid Hemorrhage,” Journal of Neuroinflammation 16, no. 1 (2019): 253, 10.1186/s12974-019-1629-7. 31801576 PMC6894125

[12] B. B. Mook‐Kanamori , M. Geldhoff , T. van der Poll , and D. van de Beek , “Pathogenesis and Pathophysiology of Pneumococcal Meningitis,” Clinical Microbiology Reviews 24, no. 3 (2011): 557–591, 10.1128/cmr.00008-11. 21734248 PMC3131058

[13] X. Tan , J. Chen , R. F. Keep , G. Xi , and Y. Hua , “Prx2 (Peroxiredoxin 2) as a Cause of Hydrocephalus After Intraventricular Hemorrhage,” Stroke 51, no. 5 (2020): 1578–1586, 10.1161/strokeaha.119.028672. 32279622 PMC7192237

[14] E. M. Massicotte and M. R. Del Bigio , “Human Arachnoid Villi Response to Subarachnoid Hemorrhage: Possible Relationship to Chronic Hydrocephalus,” Journal of Neurosurgery 91, no. 1 (1999): 80–84, 10.3171/jns.1999.91.1.0080. 10389884

[15] J. Sajant , E. Heikkinen , and K. Majamaa , “Rapid Induction of Meningeal Collagen Synthesis in the Cerebral Cisternal and Ventricular Compartments After Subarachnoid Hemorrhage,” Acta Neurochirurgica 143, no. 8 (2001): 821–826, 10.1007/s007010170036. 11678403

[16] V. Brinkmann , U. Reichard , C. Goosmann , et al., “Neutrophil Extracellular Traps Kill Bacteria,” Science (New York, N.Y.) 303, no. 5663 (2004): 1532–1535, 10.1126/science.1092385. 15001782

[17] I. Salken , J. J. Provencio , and A. P. Coulibaly , “A Potential Therapeutic Target: The Role of Neutrophils in the Central Nervous System,” Brain, Behavior, & Immunity ‐ Health 33 (2023): 100688, 10.1016/j.bbih.2023.100688. PMC10520304 37767236

[18] Q. Tan , P. Guo , J. Zhou , et al., “Targeting Neutrophil Extracellular Traps Enhanced tPA Fibrinolysis for Experimental Intracerebral Hemorrhage,” Translational Research: The Journal of Laboratory and Clinical Medicine 211 (2019): 139–146, 10.1016/j.trsl.2019.04.009. 31103467

[19] Z. Feng , L. Min , L. Liang , et al., “Neutrophil Extracellular Traps Exacerbate Secondary Injury via Promoting Neuroinflammation and Blood‐Spinal Cord Barrier Disruption in Spinal Cord Injury,” Frontiers in Immunology 12 (2021): 698249, 10.3389/fimmu.2021.698249. 34456910 PMC8385494

[20] A. D. Gregory , C. R. Kliment , H. E. Metz , et al., “Neutrophil Elastase Promotes Myofibroblast Differentiation in Lung Fibrosis,” Journal of Leukocyte Biology 98, no. 2 (2015): 143–152, 10.1189/jlb.3HI1014-493R. 25743626 PMC4763951

[21] K. Martinod , T. Witsch , L. Erpenbeck , et al., “Peptidylarginine Deiminase 4 Promotes Age‐Related Organ Fibrosis,” Journal of Experimental Medicine 214, no. 2 (2017): 439–458, 10.1084/jem.20160530. 28031479 PMC5294849

[22] S. Dimmeler and A. M. Zeiher , “Netting Insights Into Fibrosis,” New England Journal of Medicine 376, no. 15 (2017): 1475–1477, 10.1056/NEJMcibr1616598. 28402777

[23] T. Mohanty , J. Fisher , A. Bakochi , et al., “Neutrophil Extracellular Traps in the Central Nervous System Hinder Bacterial Clearance During Pneumococcal Meningitis,” Nature Communications 10, no. 1 (2019): 1667, 10.1038/s41467-019-09040-0. PMC6458182 30971685

[24] C. Pavan , A. L. R. Xavier , M. Ramos , et al., “DNase Treatment Prevents Cerebrospinal Fluid Block in Early Experimental Pneumococcal Meningitis,” Annals of Neurology 90, no. 4 (2021): 653–669, 10.1002/ana.26186. 34397111

[25] J. Li , J. P. McAllister , Y. Shen , et al., “Communicating Hydrocephalus in Adult Rats With Kaolin Obstruction of the Basal Cisterns or the Cortical Subarachnoid Space,” Experimental Neurology 211, no. 2 (2008): 351–361, 10.1016/j.expneurol.2007.12.030. 18433747

[26] Z. Chen , C. Gao , Y. Hua , R. F. Keep , K. Muraszko , and G. Xi , “Role of Iron in Brain Injury After Intraventricular Hemorrhage,” Stroke 42, no. 2 (2011): 465–470, 10.1161/strokeaha.110.602755. 21164132 PMC3078056

[27] M. Swamydas , Y. Luo , M. E. Dorf , and M. S. Lionakis , “Isolation of Mouse Neutrophils,” Current Protocols in Immunology 110 (2015): 3, 10.1002/0471142735.im0320s110. PMC4574512 26237011

[28] T. M. Hofbauer , A. Mangold , T. Scherz , et al., “Neutrophil Extracellular Traps and Fibrocytes in ST‐Segment Elevation Myocardial Infarction,” Basic Research in Cardiology 114, no. 5 (2019): 33, 10.1007/s00395-019-0740-3. 31312919 PMC6647191

[29] F. Xie , Q. Tan , A. Yu , et al., “The Role of Cell‐Free DNA in Fibrinolysis for Intraventricular Hemorrhage,” Journal of Neurosurgery 135, no. 4 (2021): 1105–1112, 10.3171/2020.7.jns201429. 33418533

[30] Z. Feng , L. Min , H. Chen , et al., “Iron Overload in the Motor Cortex Induces Neuronal Ferroptosis Following Spinal Cord Injury,” Redox Biology 43 (2021): 101984, 10.1016/j.redox.2021.101984. 33933882 PMC8105676

[31] K. Vaibhav , M. Braun , K. Alverson , et al., “Neutrophil Extracellular Traps Exacerbate Neurological Deficits After Traumatic Brain Injury,” Science Advances 6, no. 22 (2020): eaax8847, 10.1126/sciadv.aax8847. 32523980 PMC7259928

[32] Y. Wang , Y. Li , Z. Chen , et al., “GSDMD‐Dependent Neutrophil Extracellular Traps Promote Macrophage‐to‐Myofibroblast Transition and Renal Fibrosis in Obstructive Nephropathy,” Cell Death & Disease 13, no. 8 (2022): 693, 10.1038/s41419-022-05138-4. 35941120 PMC9360039

