# Supplementary material for: Neutrophil Extracellular Traps Activate Meningeal Fibroblast to Aggravate Subarachnoid Fibrosis in Kaolin‐Induced Hydrocephalus in Rats
Source: Immun Inflamm Dis. 2025 Nov 14;13(11):e70268. doi: 10.1002/iid3.70268 (PMC12616877; doi:10.1002/iid3.70268)
Supplement: Supplementary file 1 — Supplementary Fig.1: Neutrophils infiltrated from the arteries into the subarachnoid space to produce NETs. [file IID3-13-e70268-s002.docx]

**Supplemental Figure 1**

**
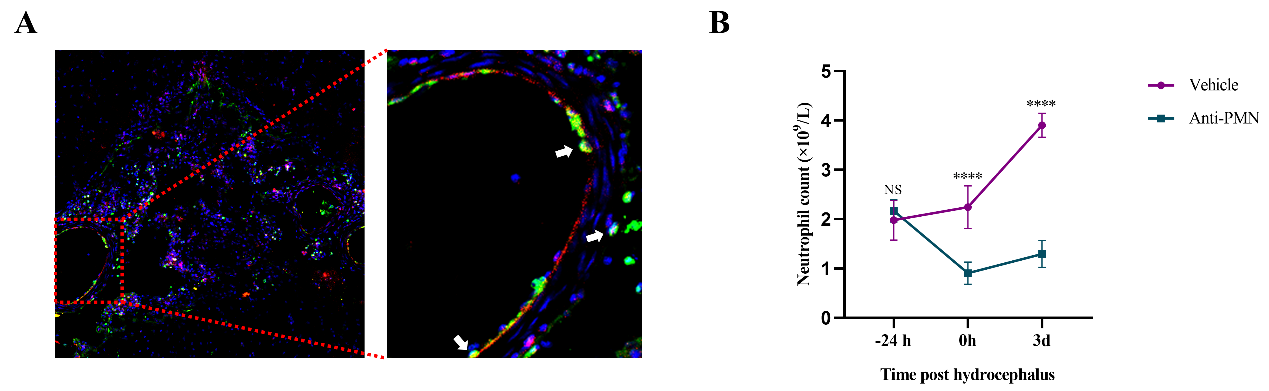
**

**Supplementary Fig.1.** Neutrophils infiltrated from the arteries into the subarachnoid space to produce NETs. (A) MPO (green) and CitH3 (red) marked citrullinated neutrophils (white arrows) infiltrated from the arteries into the subarachnoid space after after hydrocephalus induction. (B) The peripheral blood neutrophil count of vehicle and anti-PMN groups at different time points after hydrocephalus induction. Data shown as means ± SD; n = 6; NS = no significance, ****p < 0.0001.
