# Supplementary material for: Neutrophil Extracellular Traps Activate Meningeal Fibroblast to Aggravate Subarachnoid Fibrosis in Kaolin‐Induced Hydrocephalus in Rats
Source: Immun Inflamm Dis. 2025 Nov 14;13(11):e70268. doi: 10.1002/iid3.70268 (PMC12616877; doi:10.1002/iid3.70268)
Supplement: Supplementary file 2 — Table 1: Demographic and clinical characteristics of SAH patients [file IID3-13-e70268-s001.docx]

**Table 1.** Demographic and clinical characteristics of SAH patients

**SAH patient characteristics (n=14)**

**age** median (range) 57 (27 - 69)

**female** no.% 8 (57.14%)

**modified Fisher Scale** median (range) 3 (1-4)

**mRS at discharge**  median (range) 3 (0-6)
